# Is adherence to the Mediterranean diet associated with good sleep duration in primary-school children?

**DOI:** 10.3389/fped.2022.959643

**Published:** 2022-11-01

**Authors:** Alessandra Buja, Giulia Grotto, Chiara Zampieri, Simona Fortunata Mafrici, Claudia Cozzolino, Tatjana Baldovin, Filippo Brocadello, Vincenzo Baldo

**Affiliations:** ^1^ Department of Cardiological, Thoracic and Vascular Sciences, and Public Health, University of Padova, Padova, Italy; ^2^ Surgical Oncology Unit, Veneto Institute of Oncology IOV-IRCCS, Padova, Italy; ^3^ Affidea Poliambulatorio Morgagni, Padova, Italy

**Keywords:** sleep, nutrition, children, lifestyles, Mediterranean diet

## Abstract

**Objective:**

The Mediterranean diet (MD) contributes to preventing numerous chronic diseases and has benefits on cognitive development. Adherence to the MD is associated with sleep quality and duration in adults and adolescents, but this association seems to have been little investigated in primary-school children. The aim of this cross-sectional study was to verify whether good sleep duration was associated with adherence to the MD.

**Design:**

The study enrolled a sample of Italian primary school children. Their mothers were asked to answer an anonymous, self-administered questionnaire investigating the children's adherence to the MD (using the KidMed score) and variables related to their lifestyles, behavioral traits and socio-economic factors. Logistic regression models were developed to analyze the association between adherence to the MD, entered as the dependent variable, and adequacy of sleep duration.

**Setting:**

Primary schools in Padova, Italy.

**Subjects:**

267 Italian 6-year-olds in their first year of primary school.

**Results:**

The multivariate analysis showed an association between adherence to the MD and hours of sleep: for children with a good sleep duration, the odds ratio of a poor-to-moderate adherence to the MD was 0.282 (95% CI, 0.109–0.681, *p* < 0.05).

**Conclusion:**

Ensuring an adequate sleep duration may be an important strategy for enhancing adherence to the MD. Sleep and dietary education should be included in future health promotion programs.

## Statement of significance

Long sleep duration (≥10 h/day) is associated with lower odds of poor-to-moderate adherence to the Mediterranean diet (MD) in 6-year-olds children. These results suggest the importance to include sleep and dietary education in future health promotion programs.

## Introduction

The Mediterranean diet (MD) is characterized by large amounts of vegetables, whole grains, fish and dairy products, the daily consumption of olive oil, and small amounts of refined carbohydrates and animal proteins (1). It is considered one of the healthiest in the world (2). In association with other prevention measures, the MD contributes over the lifespan to reducing the risk of chronic diseases and disabilities, and premature death (3–6). A healthy diet is associated with a better cognitive and academic performance in children and adolescents (7–9). Some studies have also suggested an association between adherence to the MD and sleeping habits, in terms of sleep quality and duration (10–13). A study conducted in UK women showed that fruit and vegetable intakes differed between sleep duration categories with women sleeping the recommended 7–9 h/day having the highest intake, in cross-sectional and prospective analyses (14). Using an experimental sleep restriction protocol among adolescents, Beebe et al. found that consumption of foods with a high glycemic index (GI) and glycemic load (GL) was higher in a sleep restriction condition (<6.5 h) compared to a healthy sleep condition (∼9 h) (15), subsequently diets with a high GI, high GL, or both, increase the risk of chronic lifestyle-related diseases, such as type 2 diabetes, coronary heart disease and gallbladder disease (16). A short sleep duration has often been found related to an unbalanced diet and unhealthy lifestyle in adults and adolescents (17), but this association has not been thoroughly investigated in children. The American National Sleep Foundation recommends that school-age children sleep 9–11 h a night (18), and this amount of sleep has also been associated with better cognition in children (19). Sleep deficiency in childhood might become increasingly important as there has been a rapid, marked decline in children's average sleep duration in recent decades (20). Several studies on children have also found screen-time negatively associated with sleep quality and sleep duration (21). Meanwhile, there has been a clear tendency worldwide in recent years to move away from the MD, especially among young people (22).

Unhealthy lifestyle factors make an important contribution to an individual's modifiable disease burden, and are consequently a major public health concern (23). Understanding which factors are associated with a poor adherence to the MD may therefore be crucial to addressing the issue of unhealthy lifestyles. The aim of this study was to verify whether adherence to the MD is associated with good sleep duration in a sample of 6-year-old children.

## Materials and methods

### Participants

A project named “*Le Buone Abitudini* [Healthy Habits]” was developed to promote a varied, healthy and nutritionally-balanced diet among primary-school children in the province of Padova (north-east Italy) in the academic year 2018–2019. Of the province's 69 state primary schools, 38 were invited to take part, and 14 agreed, participating with at least one class. The present cross-sectional study derives from a survey of the mothers of children in both the participating classes and other classes at the schools involved, before the Healthy Habits project started.

**Figure 1:**
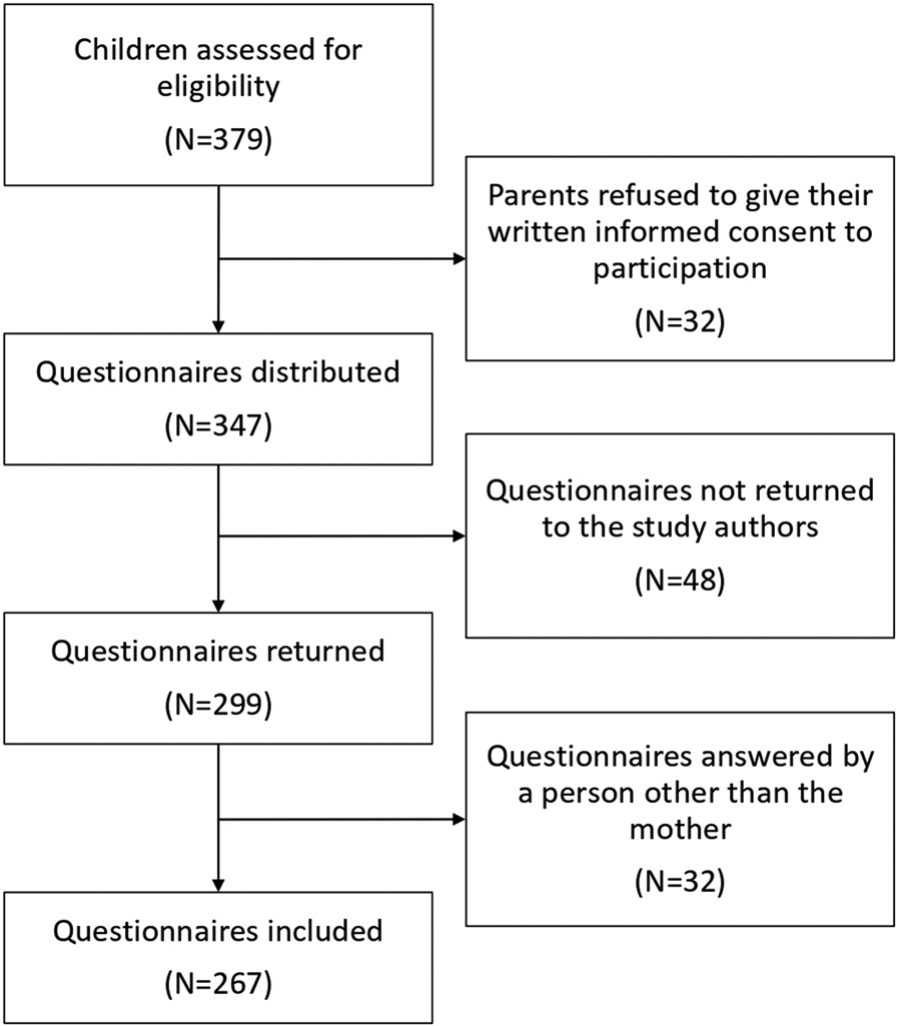
Flowchart diagram representing children lost to follow-up and included in the final analysis and the reasons for a drop-out.

The study sample concerned 379 children in their first year of primary school (6–7 years old) in 21 classes at 14 different schools. For all children in the classes involved in the project, the parents were asked to give their written informed consent to their participation. The parents of 32 children refused, and their mothers were not asked to complete the questionnaire. Of the 347 questionnaires distributed, 299 were completed and returned to the study authors, but 32 were not included in our analysis because they had been answered by a person other than the mother. In all, 267 questionnaires were thus included in this study (Figure 1).

### Materials

The children's mothers were asked to anonymously answer a self-administered questionnaire investigating several variables relating to their children's lifestyles and behavioral traits, and the family's socio-economic situation. The questionnaire contained 34 multiple-choice items and touched on all the factors potentially associated with the risk of a poor adherence to the MD, i.e., demographics, family setting, and lifestyles (24).

Children's weight and height were collected by means of the questionnaire and their body mass index (BMI) was computed using the standard formula [weight(kg)/height(m)^2^], classifying the children according to the International Obesity Task Force cut-offs, as suggested by Cole et al. (25).

The “adherence to the MD” variable was derived from the Italian version of the KidMed index (26), developed to assess the factors that sustain or undermine the adoption of Mediterranean dietary patterns. The scores range from 0 to 12, based on a test containing 16 questions (those with a negative connotation vis-à-vis the MD are assigned a value of −1, and those with a positive connotation score +1). We classified KidMed scores of 8 or more as “good”, and scores of 7 or less as “poor-to-moderate”. The validity of the measures used in this study had been tested previously, and the KidMed index has demonstrated reliable psychometric properties (27).

The “hours of sleep” variable included sleep at night and naps during the day.

Children's behavioral traits were measured on the basis of the answers their mothers gave in the Italian version of the Strengths and Difficulties Questionnaire (SDQ) (28). The SDQ uses a 3-point Likert scale (from 0 = not true to 2 = very true), and consists of five sub-scales investigating: internalizing problems, externalizing problems, hyperactivity/inattention, peer relationship problems, and prosocial behavior. Risk tertiles are identified for each behavioral trait; the first tertile identifies children showing a given behavioral trait less, and the third those who showed it more.

For lifestyle factors other than eating habits, the questionnaire investigated whether the children engaged in any competitive sports (yes or no).

The questionnaire also covered socio-economic aspects regarding the children's mothers and their family environments, including: whether they had siblings (yes or no); the mother's marital status (married or unmarried, separated, divorced or widowed); and the family's disposable income, measured with the question “How do you make ends meet with your finances?” (very/quite easily or with some/great difficulty). The mother's health literacy was tested as well, using the Italian version of the Newest Vital Sign (NVS) (29), and scoring it as “adequate” (NVS scores 4 or 5) or “marginal/limited” (NVS scores 0–3).

### Statistical methods

The proportions and 95% confidence intervals of children with a low adherence to the MD were calculated using binomial exact distribution. The associations between the adequacy of children's sleep duration (poor: <10 h, good: **≥**10 h) and the observed categorical variables were tested with Pearson's chi-squared test.

Four different logistic regression models were developed to analyze the association between poor-to-moderate adherence to the MD, entered as the dependent variable, and adequacy of sleep duration. A simple univariate model was run first (Model 1). Then multivariate logistic regressions were implemented incrementally, adjusting the effect for different covariates: the children's sex and age (Model 2); the mothers' disposable income, marital status and health literacy (Model 3); and the children's ratings in the SDQ domains (prosocial behavior, peer or conduct problems, hyperactivity, emotional symptoms), sporting activity, body mass index (BMI) and siblings (Model 4). RStudio software was used for these analyses.

### Ethical approval and consent to participation

The study was approved by the Padova Teaching Hospital's Ethical Committee (No. 4526/U6/18). The children's participation in the study was subject to the consent of the directors at the schools involved. If approved, the intervention program then became part of the school's teaching plan, which always has to be signed by parents at the start of each academic year. All parents of the children ultimately participating in the study then signed an informed consent form. All procedures also complied with the ethical standards of the Italian National Research Committee, and the 1964 Helsinki Declaration and subsequent revisions thereof, or comparable ethical standards.

## Results

Our final sample included 267 children aged 6.13 ± 0.34 years (mean ± SD), with both sexes equally represented (48.7% females) (Table 1). Only one in four children (24.3%; 95% CI, 19.32–29.95) had a good adherence to the MD, and the proportion was similar in both sexes (25.2%; 95% CI, 17.92–33.67 in females vs. 24.6%; 95% CI, 17.60–32.81 in males). The mean hours of sleep were 9.52 h per day (95% CI, 9.43–9.61), and were much the same in both sexes (9.60; 95% CI, 9.46–9.74 in females vs. 9.44; 95% CI, 9.31–9.56 in males). The bivariate distribution of the children's sleep duration by good vs. poor-to-moderate adherence to the MD showed a significant difference (*p* 0.015) in the hours of sleep between the two KidMed groups (Table 2).

**Table 1:** Characteristics of the study sample.

	Modalities	*n* (%)
**Variables concerning the child**
Sex	Females	127 (48.66%)
	Males	134 (51.34%)
Age	6 years old	231 (86.52%)
	7 years old	36 (13.48%)
Prosocial behavior	First tertile	41 (15.65%)
	Second tertile	96 (36.64%)
	Third tertile	125 (47.71%)
Peer problems	First tertile	114 (44.71%)
	Second tertile	55 (21.57%)
	Third tertile	86 (33.73%)
Hyperactivity	First tertile	28 (10.85%)
	Second tertile	143 (55.43%)
	Third tertile	87 (33.72%)
Conduct problems	First tertile	54 (21.09%)
	Second tertile	130 (50.78%)
	Third tertile	72 (28.13%)
Emotional symptoms	First tertile	72 (27.59%)
	Second tertile	122 (46.74%)
	Third tertile	67 (25.67%)
Siblings	Yes	195 (73.03%)
	No	71 (26.59%)
Sport	Yes	210 (78.65%)
	No	55 (20.60%)
BMI	<25 kg/m^2^	215 (80.52%)
	≥25 kg/m^2^	2 (0.75%)
Adherence to the MD	Good	65 (24.34%)
	Poor-to-moderate	184 (68.91%)
Sleep duration	<10 h	151 (56.55%)
	≥10 h	110 (41.20%)
**Variables concerning the mother**		
Disposable income	I make ends meet very/quite easily	168 (65.37%)
	I make ends meet with some/great difficulty	89 (34.63%)
Marital status	Married	213 (81.92%)
	Unmarried/separated /divorced/widowed	47 (18.08%)
Health literacy	Adequate	169 (74.78%)
	Marginal/limited	57 (25.22%)

**Table 2:** Bivariate analysis. Distribution of hours of sleep among primary-school children by socio-demographic and lifestyle variables.

	**Modalities**	Hours of sleep		Chi sq. test *p*
		<10 h % (*n*)	≥10 h % (*n*)	
**Variables concerning the child**				
Sex	Females	54.03 (67)	45.96 (57)	0.3126
	Males	61.07 (80)	38.93 (51)	
Age	6 years old	57.08 (129)	42.92 (97)	0.6454
	7 years old	62.86 (22)	37.14 (13)	
Prosocial behavior	First tertile	61.54 (24)	38.46 (15)	0.9211
	Second tertile	58.06 (54)	41.94 (39)	
	Third tertile	58.06 (72)	41.94 (52)	
Peer problems	First tertile	52.68 (59)	47.32 (53)	0.3642
	Second tertile	61.16 (34)	35.85 (19)	
	Third tertile	58.33 (49)	41.67 (35)	
Hyperactivity	First tertile	57.14 (16)	42.86 (12)	0.9826
	Second tertile	58.16 (82)	41.84 (59)	
	Third tertile	59.04 (49)	40.96 (34)	
Conduct problems	First tertile	53.70 (29)	46.30 (25)	0.7645
	Second tertile	57.03 (73)	42.97 (55)	
	Third tertile	60.29 (41)	39.71 (27)	
Emotional symptoms	First tertile	47.14 (33)	52.86 (37)	0.08373
	Second tertile	60.00 (72)	40.00 (48)	
	Third tertile	65.15 (43)	34.85 (23)	
Siblings	Yes	58.64 (112)	41.36 (79)	0.7101
	No	55.07 (38)	44.93 (31)	
Sport	Yes	55.77 (116)	44.23 (92)	0.272
	No	65.38 (34)	34.62 (18)	
BMI	<25 kg/m^2^	59.24 (125)	0.00 (0)	0.1695
	≥25 kg/m^2^	40.76 (86)	100.00 (2)	
Adherence to the MD	Good	44.62 (29)	55.38 (36)	0.01448
	Poor-to-moderate	63.13 (113)	36.87 (66)	
**Variables concerning the mother**				
Disposable income	I make ends meet very/quite easily	58.54 (96)	41.46 (68)	0.8385
	I make ends meet with some/great difficulty	56.32 (49)	43.68 (38)	
Marital status	Married	57.21 (119)	42.79 (89)	0.575
	Unmarried/separated/divorced/ widowed	63.04 (29)	36.96 (17)	
Health literacy	Adequate	56.02 (93)	43.98 (73)	0.4046
	Marginal/limited	63.64 (35)	36.36 (20)	

The multivariate analyses always showed an association between adherence to the MD and daily hours of sleep (Table 3): Model 1, unadjusted (OR 0.471; 95% CI, 0.263–0.834, *p* < 0.05); Model 2, adjusted for children's sex and age (OR 0.456; 95% CI, 0.253–0.816, *p* < 0.001); Model 3, adjusted for children's sex and age, and by mothers' disposable income, marital status, and health literacy (OR 0.456; 95% CI, 0.242–0.849, *p* < 0.05); Model 4, adjusted for children's sex, age, sport, BMI, siblings, and ratings in the SDQ domains, and for mothers' disposable income, marital status and health literacy (OR 0.282; 95% CI, 0.109–0.681, *p* < 0.05).

**Table 3:** Logistic multivariate models: dependent variable poor-to-moderate adherence to Mediterranean diet (KidMed score ≤7); independent variable (sleep ≥10 h/day); adjusted for different sets of covariates.

	Model 1 (unadjusted)				Model 2^a^				Model 3^b^				Model 4^c^			
	OR	2.5% CI	97.5% CI	*p*-value	OR	2.5% CI	97.5% CI	*p*-value	OR	2.5% CI	97.5% CI	*p*-value	OR	2.5% CI	97.5% CI	*p*-value
Hours of sleep ≥10 (reference <10**)**	0.471	0.263	0.834	0.010	0.456	0.253	0.816	0.008	0.456	0.242	0.849	0.014	0.282	0.109	0.681	0.006

^a^ Adjusted by child's sex and age.

^b^ Adjusted by child's sex and age, mother's disposable income, marital status and health literacy.

^c^ Adjusted by child's sex, age, BMI, sports, siblings, prosocial behavior, peer and conduct problems, hyperactivity, and emotional symptoms, and by mother's disposable income, marital status and health literacy.

## Discussion

This study aimed to assess the association between adherence to the MD and sleep duration in a sample of primary-school children, an age group in which this association has been under-investigated. We found that children who slept more had lower odds of poor-to-moderate adherence to the Mediterranean diet.

Two previous studies support the theory of a positive association between adherence to the MD and sleep duration: one performed in 2020 on 891 Portuguese children attending elementary school (30); and another conducted in Italy in 2017 on 690 greater children from 9 to 11 years old (17). In contrast, a Spanish study on 309 children aged 8–13 years found no such correlation between adherence to the MD and sleep duration (31).

Our findings suggest that the promotion of a healthy diet should also consider sleeping habits. Some dietary patterns and foods have revealed a sleep-modulating role due to the effect of certain nutrients on sleep regulation (32). Sleep has also been shown to influence eating behavior (33). Changes in sleeping habits and lack of sleep reportedly facilitate the ingestion of calories through several potential mechanisms, including: more time and opportunities for eating; psychological distress (prompting a preference for energy-dense foods); greater sensitivity to food reward; disinhibited eating; more energy needed to stay awake (34). Moreover, from a biological point of view, previous studies indicate that sleep deprivation results in changes in levels of several hormones, including leptin, ghrelin, insulin, cortisol, and growth hormone (35–37), and hormonal changes may contribute to selection of calorie-dense food, excessive food intake and changes in energy expenditure (38).

Encouraging healthy habits in children is a cost-effective strategy for promoting their cognitive development and keeping them healthy in later life—and sleep is known to have positive effects in developmental age. Magnetic resonance images of the brain of healthy 5- to 18-year-olds revealed a positive correlation between sleep duration on weekdays and the bilateral volume of the hippocampal body (39). In adolescents, sleep has shown an important role in cognitive development and mental well-being (40). A Canadian study found that children aged 7–11 who participated in a sleep education program called “Sleep for Success” extended their sleep duration and enhanced their sleep efficiency (41), with a significant improvement in their daytime functioning and academic performance (42, 43).

In conclusion, ensuring an adequate sleep duration may be an important strategy for enhancing adherence to the MD, and sleep education should be included in comprehensive healthy lifestyle promotion programs in future, alongside a healthy diet and adequate physical activity, as a relatively low-cost strategy to keep people healthy. Both healthy diet and adequate sleep could be promoted during childhood, for example through health promotion interventions in primary schools. The school setting can be an appropriate environment for assessing lifestyle, modifying misconducts, and educating students, while maintaining a stable contact with them. When aiming to improve children's sleeping and eating habits, interventions not only at school, but also in family settings have a greater likelihood of inducing behavioral changes, such as a longer sleep duration. Within this framework, schools and families are both optimal settings for programs aimed at improving children's lifestyle habits (44).

Some limitations need to be considered when interpreting the findings of the present study. For a start, the cross-sectional study design prevented us from establishing any causality for the significant association examined. It would be well worth seeking potential causal relationships and mechanisms, using appropriate study designs. Future randomized intervention trials are needed to test the effectiveness of extending children's hours of sleep in promoting their adoption of a healthier lifestyle and greater adherence to the MD. Second, our data on adherence to the MD and other variables were obtained by means of questionnaires, as we lacked the opportunity to obtain more objective evidence of the children's eating habits (such as biochemical markers). Our findings consequently suffer from the inherent limitations of self-reported data, such as the influence of social desirability. Third, the questionnaires did not aim to define a clinical diagnosis of sleep disorders or did not investigate comorbidities which can interrupt sleep duration (as rheumatoid arthritis or diabetes). Finally, another limitation of this study lies in the relatively small sample size, however it was calculated allowing to detect difference in diet adherence among sleep groups.

## Data Availability

The raw data supporting the conclusions of this article will be made available by the authors, without undue reservation.
